# Synthetic BSA-conjugated disaccharide related to the *Streptococcus pneumoniae* serotype 3 capsular polysaccharide increases IL-17A Levels, γδ T cells, and B1 cells in mice

**DOI:** 10.3389/fimmu.2024.1388721

**Published:** 2024-05-22

**Authors:** Nelli K. Akhmatova, Ekaterina A. Kurbatova, Anton E. Zaytsev, Elina A. Akhmatova, Natalya E. Yastrebova, Elena V. Sukhova, Dmitriy V. Yashunsky, Yury E. Tsvetkov, Nikolay E. Nifantiev

**Affiliations:** ^1^ Laboratory of Therapeutic Vaccines, Mechnikov Research Institute for Vaccines and Sera, Moscow, Russia; ^2^ Laboratory of Glycoconjugate Chemistry, N. D. Zelinsky Institute of Organic Chemistry, Russian Academy of Science, Moscow, Russia

**Keywords:** *Streptococcus pneumoniae* serotype 3, synthetic disaccharide, cytokine, γδ T cells, B1 Cells, interleukin 17A, antibody, mice immunoprotection

## Abstract

The disaccharide (β-D-glucopyranosyluronic acid)-(1→4)-β-D-glucopyranoside represents a repeating unit of the capsular polysaccharide of *Streptococcus pneumoniae* serotype 3. A conjugate of the disaccharide with BSA (di-BSA conjugate) adjuvanted with aluminum hydroxide induced — in contrast to the non-adjuvanted conjugate — IgG1 antibody production and protected mice against *S. pneumoniae* serotype 3 infection after intraperitoneal prime-boost immunization. Adjuvanted and non-adjuvanted conjugates induced production of Th1 (IFNγ, TNFα); Th2 (IL-5, IL-13); Th17 (IL-17A), Th1/Th17 (IL-22), and Th2/Th17 cytokines (IL-21) after immunization. The concentration of cytokines in mice sera was higher in response to the adjuvanted conjugate, with the highest level of IL-17A production after the prime and boost immunizations. In contrast, the non-adjuvanted conjugate elicited only weak production of IL-17A, which gradually decreased after the second immunization. After boost immunization of mice with the adjuvanted di-BSA conjugate, there was a significant increase in the number of CD45+/CD19+ B cells, TCR+ γδ T cell, CD5+ В1 cells, and activated cells with MHC II+ expression in the spleens of the mice. IL-17A, TCR+ γδ T cells, and CD5+ В1 cells play a crucial role in preventing pneumococcal infection, but can also contribute to autoimmune diseases. Immunization with the adjuvanted and non-adjuvanted di-BSA conjugate did not elicit autoantibodies against double-stranded DNA targeting cell nuclei in mice. Thus, the molecular and cellular markers associated with antibody production and protective activity in response to immunization with the di-BSA conjugate adjuvanted with aluminum hydroxide are IL-17A, TCR+ γδ T cells, and CD5+ В1 cells against the background of increasing MHC II+ expression.

## 1 Introduction

*Streptococcus pneumoniae* (pneumococcus) cause pneumonia, bacteremia, septic arthritis, meningitis, sinusitis, otitis media and some other diseases in humans (1, 2). The incidence of community-acquired pneumonia is one per one thousand adults. The mortality rate for pneumococcal pneumonia among hospitalized patients is 5–7% (3–7). Symptoms of pneumococcal infection depend on the localization of the infection. These may include fever, cough, chest pain, a stiff neck, chills, ear pain and others.

Pneumococcal polysaccharide and conjugate vaccines, which contain capsular polysaccharides (CPs) from clinically significant *S. pneumoniae* serotypes, are available (8). *S. pneumoniae* serotype 3 is predominant among other serotypes in various countries (9–12). Epidemiological data suggests a high incidence of disease caused by *S. pneumoniae* serotype 3 (13–15). However, the widespread use of pneumococcal vaccines should help to reduce the incidence of this disease (16–19). Improving the quality of *S. pneumoniae* type 3 in the composition of pneumococcal vaccines is essential.

Bacterial CPs contain a diverse mixture of oligosaccharides with varying chain lengths and frame shifts (20). Although their chemical preparation is practically possible (see, for example (21),), synthetic oligosaccharide derivatives represent more convenient antigenic components for the design of conjugate carbohydrate vaccines (22–25). Currently, a number of semisynthetic vaccines are under development, including those against *Staphylococcus*, *Clostridium*, *Klebsiella*, *Shigella*, and *Enterococcus* (25–33). The semi-synthetic glycoconjugate vaccine, Quimi-Hib, for the prevention of *H. influenzae* type b infection is licensed for use in Cuba (34). Optimization of the composition of pneumococcal vaccines using synthetic oligosaccharides conjugated with a protein carrier is a priority in contemporary vaccinology (25, 35–38).

Moreover, synthetic oligosaccharides with precisely defined chemical structures enable the study of the effect of bacterial antigens (39, 40), yielding a better understanding of the innate and cellular immunity, the antibody (Ab) response, and protective activity of CPs.

Immunization with glycoconjugate vaccines partially mimics the development of natural infection without actually causing the disease. In a mouse model, γδ T cells and natural killer T cells (NKT) have been shown to play a crucial role in anti-pneumococcal immunity by producing Th1 and/or Th17-related cytokines (41). The ability of semisynthetic glycoconjugates to stimulate cytokine production *in vivo* and their influence on the activation of cellular immunity remain unknown. Here, we report on the effect of a conjugate of the synthetic disaccharide, which represents a repeating unit of S. pneumoniae serotype 3 (42), on production of Th1/Th2/Th17 cytokines in mice, changes in expression of surface molecules on splenocytes, antibody response, and protection against *S. pneumoniae* infection. We also investigated the production of autoantibodies against double-stranded (ds) DNA.

## 2 Materials and methods

### 2.1 The synthetic disaccharide and its conjugate

**Figure 1:**
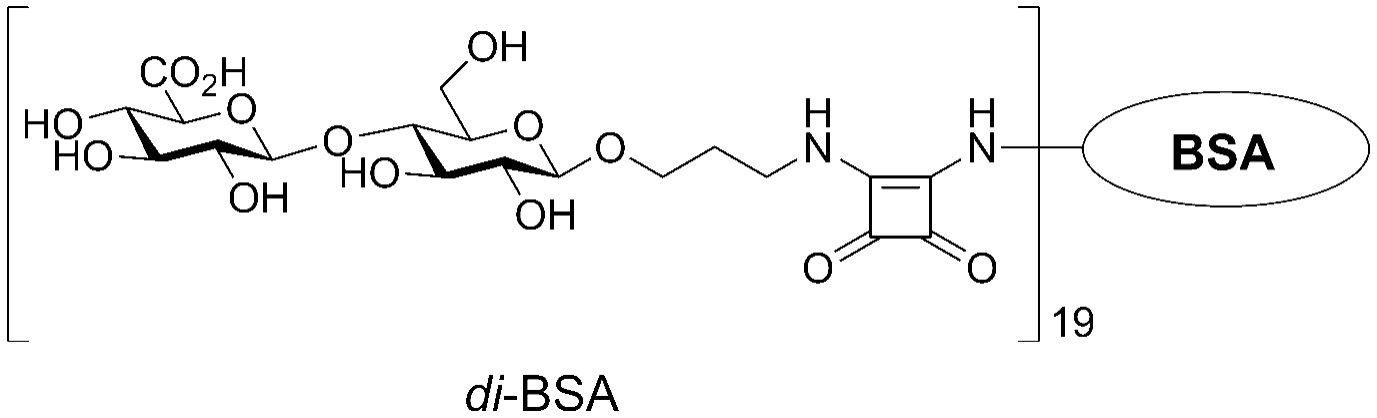
The structure of the BSA conjugate with the disaccharide that corresponds to a repeating unit of the CP from *S. pneumoniae* serotype 3.

The synthetic disaccharide (35, 43) was coupled to BSA (Sigma-Aldrich, St. Louis, MO, USA), as previously described (35, 44). The structure of the conjugate is illustrated in **Figure 1**. BSA is often used as a protein carrier in engineered immunogenic glycoconjugates and other biological systems (45). Previous studies using MALDI-TOF mass spectrometry have shown that the di-BSA conjugate contains, on average, 19 oligosaccharide ligands per protein molecule, which corresponds to a 9% carbohydrate content by weight (43, 44). The lyophilized di-BSA conjugate remains stable at +4°C, with no decrease in activity, for at least three years (i.e., observation period).

### 2.2 Bacterial capsular polysaccharide

Bacterial CP was isolated from the *S. pneumoniae* type 3 laboratory strain, #10196, which was isolated on June 30, 2011, from the blood culture of a child suffering from bacteremia in the microbiology department of the “Scientific Center for Children’s Health” in Moscow, Russia. The strain had been grown in a semi-synthetic growth medium. The isolation process for CP has been previously described elsewhere (46). The presence of CP in the preparation was confirmed by NMR spectrometry.

### 2.3 Animals

BALB/c male mice, aged 6–8 weeks (n=162), were purchased from the Scientific and Production Centre for Biomedical Technologies in Moscow, Russia, and kept in the vivarium at the Mechnikov Institute for Vaccines and Sera. Housing, breeding, blood collection, and euthanasia conditions followed European Union guidelines for laboratory animal care and use. Experimental designs were reviewed and approved (Protocol No. 2, dated February 12th, 2019) by the Ethics Committee at the Institute.

### 2.4 Conjugated disaccharide-induced cytokine production

Quantitative determination of cytokines was performed as previously described (46). Male BALB/c mice (n=6) were sacrificed, and serum was collected and stored at –20°C until further quantification of cytokine levels. Using the Flow Cytomix Mouse Th1/Th2 10-plex test system, cytokine levels were measured by adding beads coated with monoclonal antibodies to IL-1α, IL-1β, IL-2, IL-4, IL-5, IL-6, IL-10, IL-12p70, IL-13, IL-17A, IL-21 and IL-22, as well as IFNγ and TNFα, following the manufacturer’s instructions (eBioscience, San Diego, USA) using a Cytomix FC-500 flow cytometer (Beckman Coulter, Brea, USA).

### 2.5 Immunization

Mice were intraperitoneally immunized with the di-BSA conjugate, either adjuvanted or not, with aluminum hydroxide (Sigma-Aldrich). The amount of carbohydrate in 0.5 mL of the experimental semisynthetic vaccine was 20 μg, BSA ~200 μg; aluminum hydroxide, as an adjuvant, standardized for aluminum, was added in an amount of 250 μg. The single immunizing dose per mice was 0.5 mL of the di-BSA conjugate. Animals were given the vaccine twice, on days 0 and 14 of the study.

Similar immunization schedules were used for the pneumococcal conjugate vaccine Prevnar 13 (Pfizer, New York, NY, USA), which contains aluminum phosphate as an adjuvant. A 0.5 mL dose contains 2.2 μg of polysaccharides from serotypes 1, 3, 4, 5, 6A, 7F, 9V, 14, 18C, 19A, 19F, and 23F, as well as 4.4 μg of the polysaccharide from serotype 6B. The vaccine also contains 32 μg of the carrier protein, CRM_197_, and 125 μg of aluminum as aluminum phosphate. Mice were immunized twice with a single dose of 1.1 μg of CP from *S. pneumoniae* type 3 per inoculation (equivalent to half of the recommended human dose). Control mice were injected with saline.

### 2.6 Content of bacterial endotoxins in glycoconjugates

Detection of bacterial endotoxin impurities in the di-BSA conjugate was performed using the Limulus amebocyte lysate ENDOCHROME ™ (Charles River Endosafe Div. of Charles River Laboratories, Inc., Charleston, US) test obtained from the Collective Usage Center of the Mechnikov Research Institute for Vaccine and Sera (Moscow, Russia), in accordance with the manufacturer’s instructions. The di-BSA conjugate contained 0.08–0.11 EU/mL of endotoxin (LAL-Center, Moscow, Russia).

### 2.7 Measurement of antibody response to the disaccharide conjugate

Antibody titers for CP in post-immunization sera were measured using ELISA. Briefly, plates coated with *S. pneumoniae* type 3 CP were incubated with antisera from 6 immunized mice (42). Wells were washed and secondary antibody was added, followed by incubation and washing. The results were then analyzed. Enzyme substrate aliquots (100 μL) were added, followed by incubation for 20 minutes at 22°C. The reactions were quenched with 1 M H_2_SO_4_. Optical densities (ODs) were determined using an iMark microplate absorbance reader (Bio-Rad, Osaka, Japan) at a wavelength of 450 nm. Antibody titers are expressed as the dilution of serum in which the antibody was detected.

### 2.8 Expression of surface molecules on splenic mononuclear cells

Splenocytes were isolated from mice that had been vaccinated with the glyconjugate either in the absence of or in the presence of aluminum hydroxide, one and seven days after primary and booster immunizations. Single-cell suspensions of splenocytes were prepared by manually mashing the spleens using the plunger from a disposable syringe. The ground spleen was then passed through a nylon mesh and the cells were suspended in PBS. Splenic single-cell suspensions were then stained with antibodies conjugated to phycoerythrin (PE) or fluorescein isothiocyanate (FITC) to detect specific proteins in the cells: CD3e-FITC (clone 145–2C11), CD4-FITC (clone GK1.5), CD8a-FITC (clone 53–6.7), CD19-FITC (eBio1D3), CD5-PE (clone 53–7.3), NK.1.1 (clone PK136), CD3/CD16/CD32 (NKT), CD25-PE (PC61.5), CD4/CD25/Foxp3 (Treg), γδT (clone γδ TCR-PE, eBioGL3), and MHCII-PE (I-EK) (clone 14–4-45). Treg cells were stained with CD4-FITC (clone GK1.5), together with CD25-PE (PC61.5), and after fixation with the fixation/permeabilization buffer, with Foxp3- APC (clone FJK-16s). Splenocytes were incubated with 50 µL of appropriate monoclonal antibodies (eBioscience, US) at 4°C for 30 minutes. Erythrocytes were then lysed using red blood cell lysis buffer (BioLegend, US). After washing with phosphate-buffered saline (PBS), the samples were fixed using a fixation solution (BioLegend, US) and analyzed by flow cytometry (Cytomix FC-500, Beckman Coulter, USA, with the CXP software). The cell population gate was determined based on forward and side scatter and cell size. 10,000 cells were recorded per gate.

### 2.9 Di-BSA-induced active protection in immunized mice

BALB/c mice were intraperitoneally immunized with the di-BSA conjugate adsorbed or non-adsorbed on aluminum hydroxide on days 0 and 14 (twenty animals per conjugate). The same animals were intraperitoneally challenged after 2 weeks with 10^5^ colony-forming units *of S. pneumoniae* type 3/0.5 mL. Non-immunized control mice (twenty animals per conjugate) were also exposed to the bacteria. Mortality rates were determined at seven days post-infection.

### 2.10 Antibodies against double-stranded DNA

The analysis of antibodies against ds DNA in the immune sera of mice was conducted using ELISA. Salmon sperm DNA (Behringer GmbH, Germany), dissolved in a carbohydrate buffer solution at a concentration of 20 g/mL, was adsorbed onto the bottom of the wells. The plates were incubated for 2 hours at 37°C and then for additional 18 hours at 6°C. The serums were analyzed using dilutions of 1:10 to 1:1280. As secondary antibodies, secondary rabbit anti-mouse peroxidase conjugated IgG (Rockland Immunochemicals Inc., Pottstown, PA) was utilized (100 μL). After adding tetramethylbenzidine for 15 minutes, the reaction was terminated with 1 M sulfuric acid. Results were obtained utilizing a multi-channel automatic photometer (TiterTek Multiscan MC from Flow Laboratories, England), with excitation at 490nm. Serums from non-immunized mice, as well as mice immunized with either Prevnar-13 or BSA adjuvanted or non-adjuvanted with aluminum hydroxide, were used as controls.

### 2.11 Statistical analysis

Between-group comparisons were performed using Mann-Whitney rank sum tests for independent samples. Fisher exact tests were conducted to evaluate survival of mice after pneumococcal challenge. *P* values ≤0.05 were considered to indicate statistical significance. Statistical analyses were performed using the Statistical data analysis software system version 10 (StatSoft Inc., Tulsa, OK, USA).

## 3 Results

### 3.1 Antibodies induced by the di-BSA conjugate

Although the di-BSA conjugate adjuvanted with aluminum hydroxide was found to be less immunogenic than adjuvanted tri- and tetra-BSA conjugates, it was still able to induce the production of opsonizing antibodies and was sufficient for the development of serotype 3-protective immunity in mice (42).

**Figure 2:**
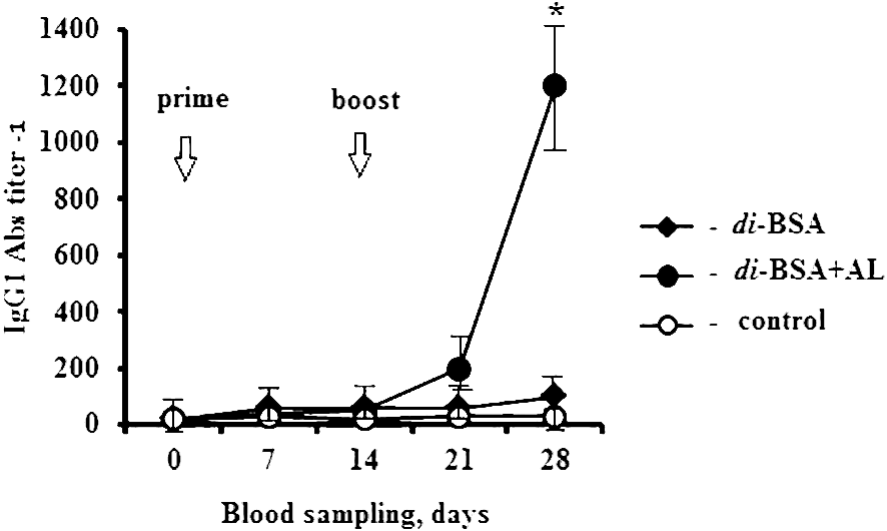
IgG1 antibody production induced by the adjuvanted and non-adjuvanted di-BSA conjugate. BALB/c mice (n = 6 per conjugate) were intraperitoneally injected with the di-BSA conjugate (20 µg/dose of carbohydrate) adjuvanted and non-adjuvanted with aluminum hydroxide, on days 0 and 14. The IgG1 Ab titer in the blood of mice was determined on days 0 (before prime immunization), days 7, 14, 21, and 28 (7 and 14 days after booster immunization, respectively), by ELISA, using CP of *S. pneumoniae* serotype 3 as the well-coating antigen. Mice (n=6) injected with saline at the same time served as a control group. AL - aluminum hydroxide. The data are presented as mean ± standard deviation (M ± SD). The Mann–Whitney rank sum test was used to determine significance, *P < 0.05.

In this study, we explored the ability of the di-BSA conjugate to induce antibodies capable of binding to the CP of *S. pneumoniae* serotype 3 in ELISA after primary and booster immunization with and without the adjuvant (**Figure 2**). The di-BSA conjugate without adjuvant did not induce Ab production after the prime and boost immunizations and no difference was observed relative to the control. The glycoconjugate adjuvanted with aluminum hydroxide induced no Ab production after prime immunization; however, after booster injection, the level of Abs increased in seven days (21 d) and was significantly elevated up to 28 d (14 d after boost). Prevnar 13 (1.1 µg/dose of carbohydrate of CP of *S. pneumoniae* serotype 3) induced IgG Ab production on day 14 after boost immunization (the time of the study) at a titer of 1:800 (data not shown).

### 3.2 Active protection upon challenge of mice immunized with the di-BSA conjugate

**Figure 3:**
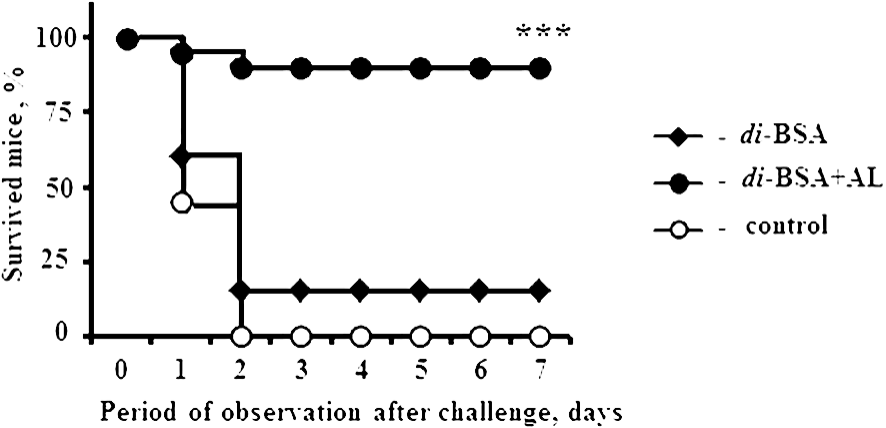
Protective activity of the adjuvanted and non-adjuvanted di-BSA conjugate. BALB/c mice (n = 20 per conjugate and control group) intraperitoneally injected with the di-BSA conjugate (20 µg/dose of carbohydrate) adjuvanted and non-adjuvanted with aluminum hydroxide on days 0 and 14 were challenged with 10^5^ colony-forming units of *S. pneumoniae* serotype 3 on day 28. Mice injected with saline were used as a control. AL - aluminum hydroxide. The results of two experiments are summarized. The difference between mice immunized with the adjuvanted di-BSA conjugate and non-adjuvanted/non-immunized mice (control) is shown. Fisher exact test; ***P < 0.001.

Mice immunized with the di-BSA conjugate and di-BSA conjugate adjuvanted with aluminum hydroxide were challenged with *S. pneumoniae* serotype 3 on day 28 (14 d after booster immunization). All control mice injected with saline and 18 out of 20 mice immunized with the non-adjuvanted di-BSA conjugate died on the second day after the challenge (**Figure 3**).

The non-adjuvanted di-BSA conjugate that failed to induce Ab production also did not elicit any protection against challenge with *S. pneumoniae* serotype 3. However, the same conjugate administered to mice with aluminum hydroxide induced protection against *S. pneumoniae* serotype 3. Thus, aluminum hydroxide is indispensable for inducing protective immunity to the disaccharide conjugate. Prevnar 13 (1.1 µg/dose of carbohydrate of CP of *S. pneumoniae* serotype 3) protected all mice (n = 6) from the challenge (42).

### 3.3 Cytokine production in mice

**Figure 4:**
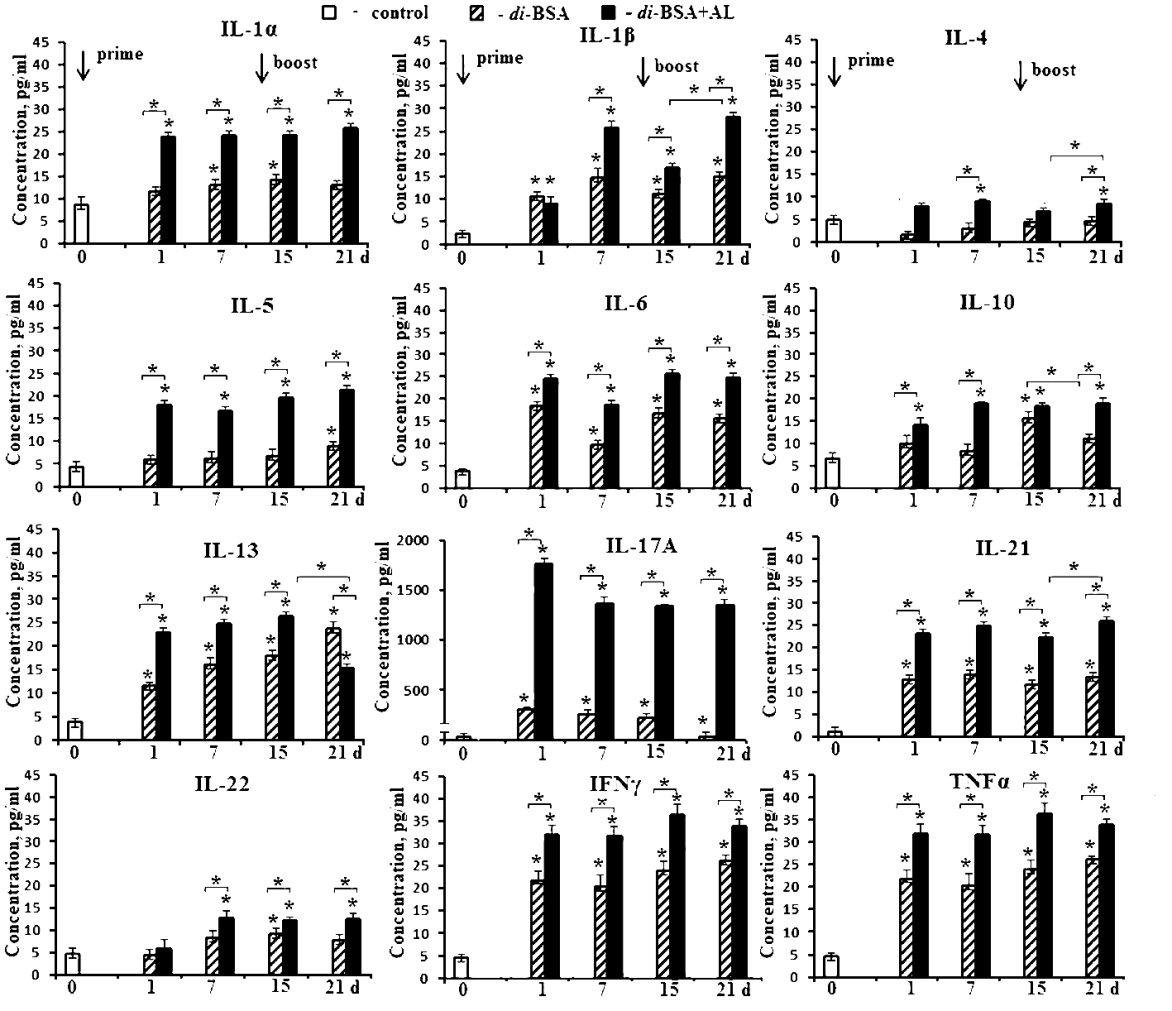
Cytokine production in mice induced by the adjuvanted and non-adjuvanted di-BSA conjugate. BALB/c mice were immunized with the di-BSA conjugate (20 μg of carbohydrate per mouse) adjuvanted or non-adjuvanted with aluminum hydroxide (n = 24 for each conjugate). Control mice (n = 6) were injected with saline 24 hours before the start of immunization (0 d). Serum was collected from mice (n=6 for each time point) after immunization. Cytokine levels were analyzed using flow cytometry. No increase in IL-2 or IL-12 p70 levels was observed in any of the time points (data not shown). The data is presented as the mean ± SD. Mann-Whitney rank sum tests were used to determine significant differences between control and other experimental groups; *P <0.05.

To evaluate cytokine production, mice were intraperitoneally injected with the di-BSA conjugate adjuvanted or non-adjuvanted with aluminum hydroxide at a single dose of 20 µg (carbohydrate content). Serum cytokine levels were determined before injection of the glycoconjugate (d 0) and on days 1, 7, 15, and 21 (1 and 7 days after boost immunization, respectively) (**Figure 4**).

After prime immunization, the non-adjuvanted di-BSA conjugate induced an increase in the levels of IL-1α, IL-1β, IL-6, IL-13, IL-17A, IL-21, IFNγ, and TNFα compared with that in the control (0 d). After booster immunization with the conjugate, IL-5, IL-10, and IL-22 production was induced in addition to these cytokines. The concentration of IL-4 did not increase in any of the study periods.

After prime immunization, the di-BSA conjugate adjuvanted with aluminum hydroxide stimulated higher production of IL-1α, IL-1β, IL-4, IL-5, IL-6, IL-10, IL-13, IL-17A, IL-21, IL-22, IFNγ, and TNFα compared with the conjugate without the adjuvant. After booster immunization, all cytokines were found to be produced at high levels. When the conjugate was administered with the adjuvant, a very high level of IL-17A production was noted at all time points. In contrast, when mice were immunized with the conjugate without the adjuvant, the IL-17A level gradually decreased even after booster immunization. Regardless of the presence of the adjuvant, the levels of IL-2 and IL-12p70 did not increase during all follow-up periods. Free CP of *S. pneumoniae* serotype 3 (5 µg/mouse) elevated only the level of IFNγ (from 23.1 to 50.8 pg) after double immunization (data not shown). CP-CRM_197_ (Prevnar 13) is able to induce the production of IL-1, IL-2, IL-4, IL-5, IL-6, IL-10, IL-12, IL-17, IFNγ, and TNFα (47, 48). Free aluminum hydroxide did not elicit cytokine production when administered at the same time points (data not shown).

### 3.4 Expression of cell-surface molecules on splenic mononuclear cells

**Figure 5:**
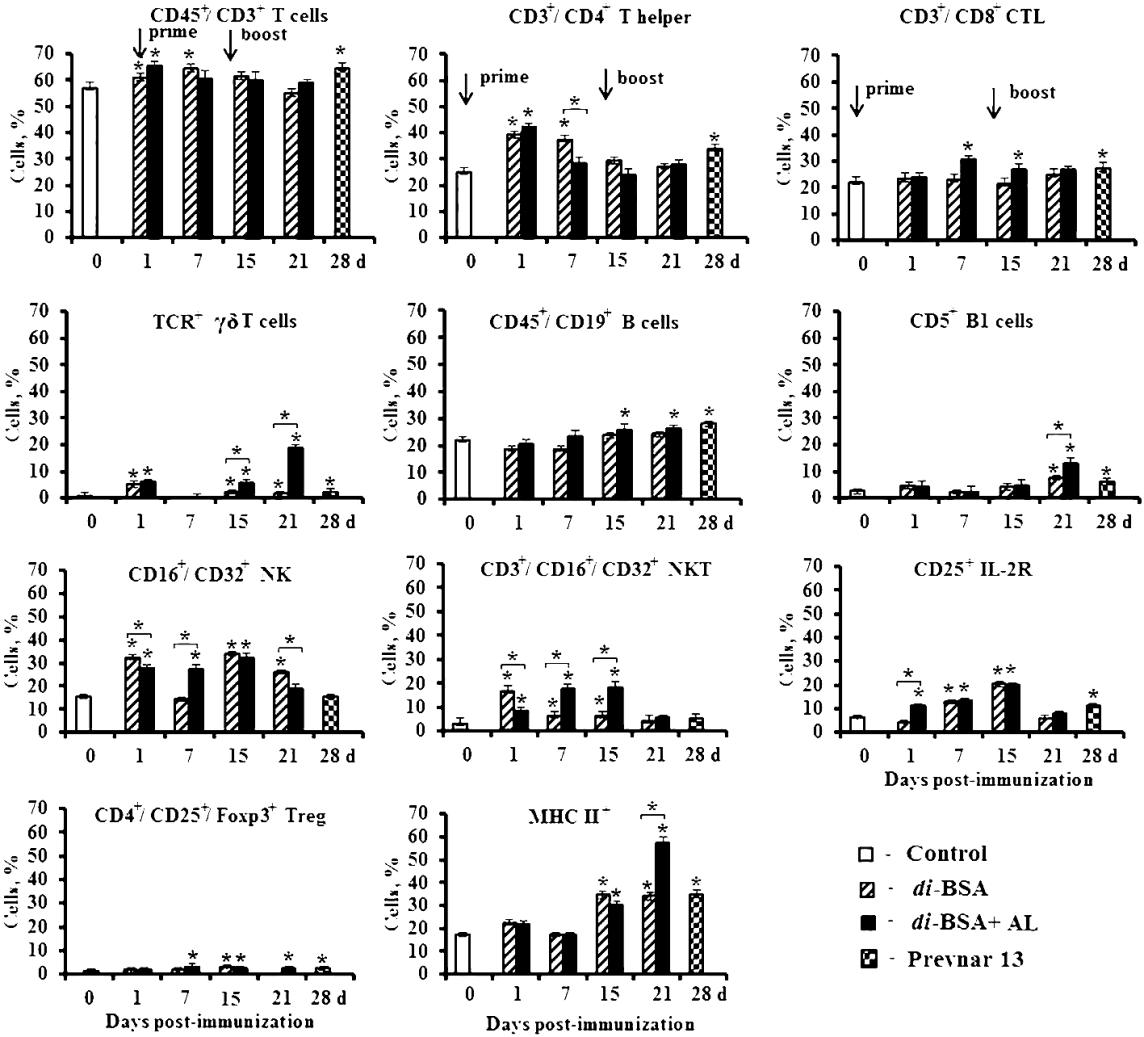
The number of splenocytes expressing membrane molecules in mice immunized with the di-BSA conjugate with and without adjuvant. BALB/c mice were immunized with the di-BSA conjugate (20 μg/dose of carbohydrate per mouse) adjuvanted or non-adjuvanted with aluminum hydroxide and with Prevnar 13 (1.1 μg/dose of carbohydrate of CP *S. pneumoniae* serotype 3 per mouse) adjuvanted with aluminum phosphate. Splenocytes were isolated from mice (n = 6 for each conjugate and each time point) on the indicated days after immunization. Control mice (n = 6) were injected with saline 24 hours before the start of immunization (0 d). Spleen cell suspensions were stained using antibodies against mouse CD3e-FITC (clone 145–2C11), CD4-FITC (clone GK1.5), CD8a-FITC (clone 53–6.7), γδT (clone γδ TCR-PE, eBioGL3), CD19-FITC (eBio1D3), CD5-PE (clone 53–7.3), NK.1.1 (clone PK136) CD25-PE (PC61.5), and MHCII-PE (I-EK) (clone 14–4-45). Treg: FITC anti-mouse CD4 (clone GK1.5). Staining with anti-Foxp3-APCconjugated Ab (clone FJK-16s) was performed according to the manufacturer’s protocol. The results were determined using flow cytometry. The data are shown as the mean ± SD. Mann-Whitney rank sum tests were used to calculate significant differences between control and other experimental groups; *P < 0.05.

After first immunization with the di-BSA conjugate adjuvanted and non-adjuvanted with aluminum hydroxide, the number of CD45^+^/CD3^+^ T cells and CD45^+^/CD4^+^ T helper cells increased compared with that in the control. After booster immunization, regardless of the presence of adjuvant, there was no difference relative to the control (**Figure 5**).

After primary and booster immunization with the adjuvanted di-BSA conjugate, the number of CD45^+^/CD8^+^ cytotoxic T cells (CTLs) increased compared with that in the control. The non-adjuvanted conjugate did not induce any change in the number of CTLs during the entire observation period. An interesting result was revealed in relation to γδ T cells. One day after prime immunization of mice with the adjuvanted and non-adjuvanted di-BSA conjugate, the number of γδ T cells increased compared with that in the control and decreased to the initial levels on day 7. However, after booster immunization with the adjuvanted conjugate, the number of TCR^+^ γδ T cells increased on day 15 (1 d after boost), reaching high values on day 21 (7 d after boost). In contrast, in the absence of aluminum hydroxide, their values did not differ from the control level. After booster immunization with the di-BSA conjugate, the number of CD45^+^/CD19^+^ B cells increased only following booster immunization in the presence of aluminum hydroxide. After injection of the non-adjuvanted conjugate, the level of CD45^+^/CD19^+^ B cells did not differ from that in the control. The number of CD5^+^ B1 increased on day 1 after the first immunization with adjuvanted and non-adjuvanted conjugate compared with that in the control and then decreased on day 7. Booster immunization with the adjuvanted di-BSA conjugate led to an increase of number of CD5^+^ B1 cells on day 15 (1 d after boost) compared with that in the control, and on day 21 (7 d after boost) relative to the non-adjuvanted conjugate. The administration of the adjuvanted and non-adjuvanted conjugate increased the number of CD16^+^/CD32^+^ natural killer cells (NK) and CD3^+^/CD16^+^/CD32^+^ natural killer T cells (NKT) after primary and booster immunization. The adjuvanted and non-adjuvanted di-BSA conjugate led to increase in the number of cells expressing CD25+ and the IL-2 receptor and CD4^+^/CD25^+^/Foxp3^+^ T regulatory cells (Treg). The number of cells expressing MHC II^+^ increased only after booster immunization—to a greater extent on day 21 (7 d after boost)—and was higher than that in the case of conjugate administration without aluminum hydroxide.

Prevnar 13, containing a CRM_197_-CP of *S. pneumoniae* serotype 3 conjugate, induced similar changes on day 28 (14 d after booster immunization) in the number of cells expressing cell-surface molecules. Specifically, there was an increased number of (TCR^+^) γδ T cells, CD45^+^/CD8^+^ CTLs, CD5^+^ B1 cells, CD45^+^/CD19^+^ B cells, CD4^+^/CD25^+^/Foxp3^+^ Tregs, cells expressing CD25^+^, and cells expressing MHC II+. The number of NK- and NKT-cells did not differ from that in the control.

An elevation in Ab production and protection against *S. pneumoniae* serotype 3 was detected only after double immunization with the adjuvanted di-BSA conjugate. This finding suggests that the cells whose number showed a large increase after booster immunization (TCR^+^ γδT cells and CD5^+^ B1 cells), against the background of an increase in the number of activated cells expressing MHC II^+^, play a crucial role in the protective activity of the conjugate.

### 3.5 Antibodies against double-stranded DNA

**Figure 6:**
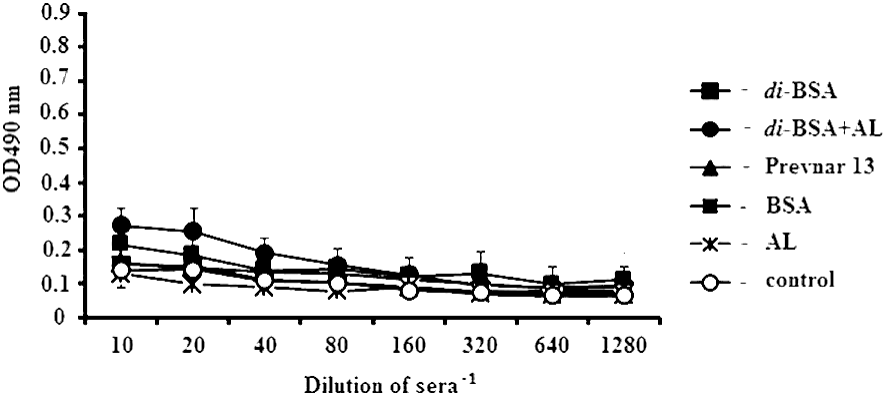
IgG antibodies to double-stranded DNA in immunized mice, analyzed by ELISA. ds DNA was used as the well-coating antigen. Sera to each conjugate, BSA, aluminum hydroxide, and control (non-immunized mice) (n = 6 for each antigen) was added to each well in dilutions from 1:10 to 1:1280. AL - aluminum hydroxide; control - mice injected with saline. After prime-boost immunization, autoantibodies to ds DNA, which target the cell nuclei, were not detected.

No difference was observed in the level of Abs against ds DNA relative to the control at the dilution of 1:80 in sera of mice immunized with the di-BSA conjugate adsorbed and non-adsorbed on aluminum hydroxide, Prevnar 13, BSA, and free aluminum hydroxide (**Figure 6**).

## 4 Discussion

In contrast to the conjugate without adjuvant, the di-BSA conjugate adjuvanted with aluminum hydroxide, induced production of IgG1 antibodies and protected mice against *S. pneumoniae* serotype 3 after prime-boost immunization. The role of adjuvants in enhancing the adaptive immune response to antigens, including semisynthetic glycoconjugates corresponds to the data of other authors (49–51).

The concentrations of IL-1α, IL-1β, IL-4, IL-5, IL-6, IL-10, IL-13, IL-17A, IL-21, IL-22, IFNγ, and TNFα in mice sera in response to the di-BSA conjugate adjuvanted with aluminum hydroxide were higher compared with those in response to the non-adjuvanted glycoconjugate. Free aluminum hydroxide is known to stimulate the production of IL-1β and IL-18, and, when administered with antigens, the spectrum of cytokines expands (52–58). IFNγ, IL-17A, and IL-22 (a member of the Th17 cytokine family) plays a role in the early stages of controlling *S. pneumoniae* infections (59–67). IL-17 has an important function in protecting against bacterial carriage and lung infection (59, 65, 68–71). The di-BSA conjugate adjuvanted with aluminum hydroxide induced a very high level of IL-17A after the prime and boost immunizations, while the conjugate without adjuvant caused a weak production of IL-17A that gradually decreased after the booster injection. A high level of Th2 cytokines (IL-4 and IL-5) was revealed in mice immunized with the adjuvanted di-BSA conjugate. Th2 cytokines promote switching from IgM to IgG, which is associated with high production of IgG1 antibodies (72). The conjugate without adjuvant did not elicit IL-4 production, only weakly stimulated the production of IL-5 even after boost immunization, and did not induce the antibody response. Prevnar 13 is known to induce the production of Th1/Th2 and Th17 cytokines (47, 48). In our previous studies, we have shown that Prevnar 13 induced anti-CP *S. pneumoniae* type 3 IgG1-antibodies and protected immunized mice from the challenge with *S. pneumoniae* type 3 (42).

The di-BSA conjugate and CPs, including that of *S. pneumoniae* serotype 3, are not Toll-like receptor (TLR) ligands (46). Purified CP from *S. pneumoniae* can bind to macrophages through the carbohydrate-recognition domains on the mannose receptor, leading to the production of proinflammatory cytokines such as IL-1, IL-6, and TNFα, as well as chemokines (73). Another receptor, the C-type lectin, also known as carbohydrate-binding protein, SIGN-R1, is expressed by macrophages, particularly in the marginal zone of the mouse spleen. This receptor is able to bind carbohydrates from several different serotypes of *S. pneumoniae* (73). Other carbohydrate-recognition receptors of macrophages remain to be identified (74). It is likely that macrophages play a significant role in the initial stage of the immune response to the di-BSA conjugate (36, 59, 74–77).

Regardless of the presence of the adjuvant, the number of CD4^+^ T helper cells involved in the adaptive immune response to the antigen increased only after the first immunization with the di-BSA conjugate. The number of CD4^+^ T cells after booster immunization with the BSA-conjugated synthetic hexasaccharide related to *S. pneumoniae* serotype 14 CP adsorbed on aluminum hydroxide did not differ from that in the control either (46). However, the number of CD4^+^ T helper cells increased on day 14 after booster immunization in mice immunized with CP of *S. pneumoniae* serotype 3 conjugated to CRM_197_ and adsorbed on aluminum phosphate (Prevnar 13). This result may be attributable to the multicomponent composition of the vaccine and the presence of a small amount of bacterial impurities remaining even after purification of CPs. The number of CD8^+^ cytotoxic cells (CTL) in response to the disaccharide conjugate and Prevnar 13 increased.

Both the adjuvanted di-BSA conjugate and Prevnar 13 significantly increased the number of (TCR^+^) γδ T cells among the splenocytes after booster immunization. γδ T cells play a crucial role in prevention of pneumococcal infection owing to their ability to recognize unprocessed non-peptide antigens (41). A large number of γδ T cell ligands remain unknown to date (78, 79). In mice, most γδ T cells are found in the body’s barrier tissues, with a small proportion in the blood and spleen (46, 80–83). The activation of γδ T cells through TCRs can be mediated by non-classical MHC molecules (e.g., T10/T22 and members of the CD1 family) and MHC-unrelated molecules (e.g., viral glycoproteins and butyrophilin 3A1) (79, 84–87). Putatively, γδ T cells bind the oligosaccharide portion of the glycoconjugate without processing in antigen-presenting cells (APCs) in combination with MHC-like molecules activate cytokine production. γδ T cells produce a large variety of cytokines and exhibit potent cytotoxic activity against pathogens through apoptosis-inducing receptors (FAS and TRAIL), as well as cytolytic proteins such as perforin and granzyme (88, 89). Furthermore, γδ T cells can function as professional APCs that require surface interactions with opsonized cells (90). The di-BSA conjugate has been shown to induce the formation of opsonizing antibodies (42). Certain subsets of γδ T cells express CD4. These cells have a Th1 or Th2 phenotype and produce IL-2, IL-4, IL-17A, IFNγ, and TNF (70). The di-BSA conjugate induced the production of IFNγ and TNFα (Th1 cytokines); IL-4, IL-5, and IL-13 (Th2 cytokines); IL-17A (Th17 cytokines); IL-21 (Th2 and Th17 subsets); and IL-22 (Th1 and Th17 subsets). γδ T cells play a crucial role in immune protection against extracellular respiratory bacteria (41, 91, 92). The potential role of γδ T cells in pneumococcal infection has only been investigated in animal models using *S. pneumoniae* serotype 3 (41). During infection, the number of γδ T cells can significantly increase, accounting for up to 50% of all peripheral lymphocytes (93, 94). In the mouse model, γδ T cells accumulate and become activated in the lungs during *S. pneumoniae* infection (95, 96). Mice with a lack of γδ T cells exhibit a higher bacterial load in their lungs and lower survival rates compared to control mice (66, 95, 97). The absence of γδ T cells is associated with impaired secretion of MIP-2, TNFα, and IL-17, as well as a poor recruitment of neutrophils (66, 95, 97). In addition, γδ T cells produce IFNγ during *S. pneumoniae* infections of serotypes 3 and 1. Along with their early role in defense against *S. pneumoniae*, γδ T cells participate in the resolution stage of pneumococcal pneumonia, eliminating inflammatory mononuclear phagocytes (98). Therefore, γδ T cells are essential for the host’s defense against *S. pneumoniae* (66, 95).

The di-BSA conjugate adjuvanted with aluminum hydroxide and Prevnar 13 adsorbed on aluminum phosphate induced a significant increase CD5^+^ B1 cells after booster immunization. CD5^+^ B1 cells are mainly located in the peritoneal and pleural cavities, but very small amounts were also found in the spleen (99, 100). CD5^+^ В1 cells are activated primarily by T-independent antigens (101, 102) and play an important role in protecting against pneumococcal infections (103) This role may be attributed to their production of natural antibodies as well as possible participation in the T-dependent immune response (102, 104–109). The B cell receptor (BCR) is involved in the phagocytosis of bacteria by B1 cells (110). CD5^+^ B1, isolated from the spleens of mice, primarily induce IL-17 production by T cells (111). B1 cells present antigen to antigen-specific T cells and induce more efficient proliferation than conventional CD19^+^ B cells (107, 108). After immunization with the di-BSA conjugate, the number of CD19^+^ B cells in the blood increased, regardless the presence of the adjuvant. The number of CD19^+^ B cells increased during all observation periods. Ovalbumin-presenting B1 cells were found to express a higher level of MHC class II compared to naïve B1 cells.

Immunization with either the adjuvanted or the non-adjuvanted di-BSA conjugate increases the number of natural killer (NK) cells and natural killer T (NKT) cells. NK cells, through the production of IFNγ, participate in the early immune response to pulmonary *S. pneumoniae* infection. NKT cells have a key role in protecting against pneumococcal infection. When mice lacking NKT cells were infected with *S. pneumoniae* serotype 3, they exhibited a higher mortality rate and bacterial load in their lungs compared to wild-type mice. It has been suggested that IFNγ derived from NKT cells has a critical function in protecting mice against pneumococcal pneumonia. Using *S. pneumoniae* serotype 1, it has also been found that NKT cells are an important innate immune effector in clearing pneumococci from the body. NKT cells can indirectly or directly assist B cells in mounting antibody responses and have a crucial role in the production of antibodies against pneumococcus and in the switch of classes in response to the administration of pneumococcal vaccines (112–114).

IL-17A, γδ T, and CD5^+^ В1 cells can also contribute to autoimmune diseases (115). In response to infection or immunization, autoreactive clones of B1 cells can be produced in the body’s own tissues (109, 116–118). The expansion of autoreactive clones of B cells is controlled by IL-10, leaving the BCR in a state of anergy. After booster immunization, there was an increase in the number of CD4^+^/CD25^+^/FoxP3^+^ T regulatory cells (Tregs) on the background of interleukin-10 (IL-10) production, which regulates the development of the immune response. After prime-boost immunization with the di-BSA conjugate or Prevnar 13, no formation of autoantibodies against ds DNA targeting cell nuclei was detected.

## 5 Conclusion

The key effectors of the immune response in mice following immunization with aluminum hydroxide adjuvanted di-BSA conjugate, associated with antibody response and protection from infection by *S. pneumoniae* serotype 3, were IL-17A, γδ T, and CD5^+^ B1 cells, with an increase in the number of MHC II-expressing cells after booster immunization. The roles of non-conventional γδ T cells, B1 cells, and production of IL-17A upon pneumococcal immunization with the semisynthetic glycoconjugate may provide an in-depth understanding of post-vaccination defense mechanisms, enabling the development of novel efficient therapies and improvement of existing vaccine formulations.

## Data availability statement

The original contributions presented in the study are included in the article/supplementary material. Further inquiries can be directed to the corresponding author/s.

## Ethics statement

The animal study was approved by Mechnikov Research Institute for Vaccines and Sera Ethics Committee. The study was conducted in accordance with the local legislation and institutional requirements.

## Author contributions

EK: Conceptualization, Investigation, Writing – original draft. NA: Investigation, Methodology, Writing – review & editing. AZ: Investigation, Methodology, Writing – review & editing. EA: Investigation, Methodology, Writing – review & editing. NY: Investigation, Methodology, Writing – review & editing. ES: Investigation, Methodology, Writing – review & editing. DY: Investigation, Methodology, Writing – review & editing. YT: Investigation, Methodology, Writing – review & editing. NN: Conceptualization, Funding acquisition, Project administration, Writing – review & editing.

## References

[1] Rodgers GL Arguedas A Cohen R Dagan R. Global serotype distribution among *Streptococcus pneumoniae* isolates causing otitis media in children: potential implications for pneumococcal conjugate vaccines. Vaccine. (2009) 27:3802–10. doi: 10.1016/j.vaccine.2009.04.021 19446378

[2] O'Brien KL Wolfson LJ Watt JP Henkle E Deloria-Knoll M McCall N. Burden of disease caused by *Streptococcus pneumoniae* in children younger than 5 years: global estimates. Lancet. (2009) 374:893–902. doi: 10.1016/S0140-6736(09)61204-6 19748398

[3] Martens P Worm SW Lundgren B Konradsen HB. Benfield T Serotype-specific mortality from invasive *Streptococcus pneumoniae* disease revisited. BMC Infect Dis. (2004) 4:21. doi: 10.1186/1471-2334-4-21 15228629 PMC455681

[4] Harboe ZB Thomsen RW Riis A Valentiner-Branth P Christensen JJ Lambertsen L. Pneumococcal serotypes and mortality following invasive pneumococcal disease: a population-based cohort study. PLoS Med. (2009) 6:e1000081. doi: 10.1371/journal.pmed.1000081 19468297 PMC2680036

[5] Weinberger DM Harboe ZB Sanders EA Ndiritu M Klugman KP Rückinger S. Association of serotype with risk of death due to pneumococcal pneumonia: a meta-analysis. Clin Infect Dis. (2010) 51:692–9. doi: 10.1086/655828 PMC2927802 20715907

[6] Grabenstein JD Musey LK. Differences in serious clinical outcomes of infection caused by specific pneumococcal serotypes among adults. Vaccine. (2014) 32:2399–405. doi: 10.1016/j.vaccine.2014.02.096 24637174

[7] Inverarity D Lamb K Diggle M Robertson C Greenhalgh D Mitchell TJ. Death or survival from invasive pneumococcal disease in Scotland: associations with serogroups and multilocus sequence types. J Med Microbiol. (2011) 60:793–802. doi: 10.1099/jmm.0.028803-0 21393453 PMC3167921

[8] PLoSker GL. 13-valent pneumococcal conjugate vaccine: a review of its use in infants, children, and adolescents. Pediatr Drugs. (2013) 15:403–23. doi: 10.1007/s40272-013-0047-z 24030738

[9] Namkoong H Ishii M Funatsu Y Kimizuka Y Yagi K Asami T. Theory and strategy for pneumococcal vaccines in the elderly. Hum Vaccin Immunother. (2016) 12:336–43. doi: 10.1080/21645515.2015.1075678 PMC5049722 26406267

[10] Gransden WR Eykyn SJ Phillips I. Pneumococcal bacteraemia: 325 episodes diagnosed at St Thomas’s Hospital. Br Med J (Clin Res Ed). (1985) 290:505–8. doi: 10.1136/bmj.290.6467.505 PMC1417989 3918650

[11] Inostroza J Vinet AM Retamal G Lorca P Ossa G Facklam RR. Influence of patient age on *Streptococcus pneumoniae* serotypes causing invasive disease. Clin Diagn Lab Immunol. (2001) 8:556–9. doi: 10.1128/CDLI.8.3.556-559.2001 PMC96100 11329457

[12] Scott JA Hall AJ Dagan R Dixon JM Eykyn SJ Fenoll A. Serogroup-specific epidemiology of *Streptococcus pneumoniae*: associations with age, sex, and geography in 7,000 episodes of invasive disease. Clin Infect Dis. (1996) 22:973–81. doi: 10.1093/clinids/22.6.973 8783696

[13] España PP Uranga A Ruiz LA Quintana JM Bilbao A Aramburu A. Evolution of serotypes in bacteremic pneumococcal adult pneumonia in the period 2001–2014, after introduction of the pneumococcal conjugate vaccine in Bizkaia (Spain). Vaccine. (2019) 37:3840–8. doi: 10.1016/j.vaccine.2019.05.052 31153692

[14] Lee S Lee K Kang Y Bae S. Prevalence of serotype and multidrug-resistance of S. pneumoniae respiratory tract isolates in 265 adult and 36 children in Korea, 2002–2005. Microb Drug Resist. (2010) 16:135–42. doi: 10.1089/mdr.2009.0114 20370508

[15] Jansen AG Hak E Veenhoven RH Damoiseaux RA Schilder AG Sanders EA. Pneumococcal conjugate vaccines for preventing otitis media. Cochrane Database Syst Rev. (2009) 2:CD001480. doi: 10.1002/14651858.CD001480.pub3 19370566

[16] Shiramoto M Hanada R Juergens C Shoji Y Yoshida M Ballan B. Immunogenicity and safety of the 13-valent pneumococcal conjugate vaccine compared to the 23-valent pneumococcal polysaccharide vaccine in elderly Japanese adults. Hum Vaccin Immunother. (2015) 11:2198–206. doi: 10.1080/21645515.2015.1030550 PMC4635730 26176163

[17] Andrews NJ Waight PA Burbidge P Pearce E Roalfe L Zancolli M. Serotype-specific effectiveness and correlates of protection for the 13-valent pneumococcal conjugate vaccine: a postlicensure indirect cohort study. Lancet Infect Dis. (2014) 14:839–46. doi: 10.1016/S1473-3099(14)70822-9 25042756

[18] Schuerman L Prymula R Henckaerts I Poolman J. ELISA IgG concentrations and opsonophagocytic activity following pneumococcal protein D conjugate vaccination and relationship to efficacy against acute otitis media. Vaccine. (2007) 25:1962–8. doi: 10.1016/j.vaccine.2006.12.008 17258357

[19] Prymula R Peeters P Chrobok V Kriz P Novakova E Kaliskova E. Pneumococcal capsular polysaccharides conjugated to protein D provide protection against otitis media caused by both *Streptococcus pneumoniae* and non-typable *Haemophilus influenzae*: a randomized double blind efficacy study. Lancet. (2006) 367:740–8. doi: 10.1016/S0140-6736(06)68304-9 16517274

[20] Yu X Sun Y Frasch C Concepcion N Nahm MH. Pneumococcal capsular polysaccharide preparations may contain non-C-polysaccharide contaminants that are immunogenic. Clin Diagn Lab Immunol. (1999) 6:519–24. doi: 10.1128/CDLI.6.4.519-524.1999 PMC95719 10391854

[21] Kochetkov NK Nifant'ev NE Backinowsky LV. Synthesis of the capsular polysaccharide of *Streptococcus pneumoniae* type 14. Tetrahedron. (1987) 43:3109–21. doi: 10.1016/S0040-4020(01)86852-6 3580006

[22] Sorieul C Dolce M Romano MR Codée J Adamo R. Glycoconjugate vaccines against antimicrobial resistant pathogens. Expert Rev Vaccines. (2023) 22:1055–78. doi: 10.1080/14760584.2023.2274955 37902243

[23] del Bino L Østerlid KE Wu D-Y Nonne F Romano MR Codée J. Synthetic glycans to improve current glycoconjugate vaccines and fight antimicrobial resistance. Chem Rev. (2022) 122:15672–716. doi: 10.1021/acs.chemrev.2c00021 PMC9614730 35608633

[24] Krylov VB Nifantiev NE. Synthetic carbohydrate based anti-fungal vaccines. Drug Discovery Today: Technol. (2020) 35–36:35–43. doi: 10.1016/j.ddtec.2020.11.002 33388126

[25] Anish C Schumann B Pereira CL Seeberger PH. Chemical biology approaches to designing defined carbohydrate vaccines. Chem Biol. (2014) 21:38–50. doi: 10.1016/j.chembiol.2014.01.002 24439205

[26] Gening ML Maira-Litran T Kropec A Skurnik D Grout M Tsvetkov YE. Synthetic β(1→6)-linked N-acetylated and non-acetylated oligoglucosamines used to produce conjugate vaccines for bacterial pathogens. Infect Immun. (2010) 78:764–72. doi: 10.1128/IAI.01093-09 PMC2812210 19948836

[27] Parameswarappa SG Reppe K Geissner A Ménová P Govindan S Calow ADJ. A semi-synthetic oligosaccharide conjugate vaccine candidate confers protection against *Streptococcus pneumoniae* serotype 3 infection. Cell Chem Biol. (2016) 23:1407–16. doi: 10.1016/j.chembiol.2016.09.016 PMC5234679 27818299

[28] Seeberger PH Pereira CL Khan N Xiao G Diago-Navarro E Reppe K. A semi-synthetic glycoconjugate vaccine candidate for Carbapenem-resistant *Klebsiella pneumoniae* . Angew Chem Int Ed Eng. (2017) 56:13973–8. doi: 10.1002/anie.201700964 PMC5819008 28815890

[29] Broecker F Hanske J Martin CE Baek JY Wahlbrink A Wojcik F. Multivalent display of minimal *Clostridium difficile* glycan epitopes mimics antigenic properties of larger glycans. Nat Commun. (2016) 7:11224. doi: 10.1038/ncomms11224 27091615 PMC4838876

[30] Broecker F Martin CE Wegner E Mattner J Baek JY Pereira CL. Synthetic lipoteichoic acid glycans are potential vaccine candidates to protect from *Clostridium difficile* infections. Cell Chem Biol. (2016) 23:1014–22. doi: 10.1016/j.chembiol.2016.07.009 27524293

[31] Solovev AS Denisova EM Kurbatova EA Kutsevalova OY Boronina LG Ageevets VA. Synthesis of methylphosphorylated oligomannosides structurally related to lipopolysaccharide O-antigens of *Klebsiella pneumoniae* serotype O3 and their application for detection of specific antibodies in rabbit and human sera. Org Biomol Chem. (2023) 21:8306–19. doi: 10.1039/D3OB01203D 37794804

[32] van der Put RMF Smitsman C de Haan A Hamzink M Timmermans H Uittenbogaard J. The first-in-human synthetic glycan-based conjugate vaccine candidate against *Shigella* . ACS Cent Sci. (2022) 8:449–60. doi: 10.1021/acscentsci.1c01479 PMC9088300 35559427

[33] Laverde D Romero-Saavedra F Argunov DA Enotarpi J Krylov VB Kalfopoulou E. Synthetic Oligomers Mimicking Capsular Polysaccharide Diheteroglycan are Potential Vaccine Candidates against Encapsulated Enterococcal Infections. ACS Infect Dis. (2020) 6:1816–26. doi: 10.1021/acsinfecdis.0c00063 32364376

[34] Aguilar-Betancourt A González-Delgado CA Cinza-Estévez Z Martínez-Cabrera J Véliz-Ríos G Alemán-Zaldívar R. Safety and immunogenicity of a combined hepatitis B virus-*Haemophilus influenzae* type B vaccine comprising a synthetic antigen in healthy adults. Hum Vaccin. (2008) 4:54–9. doi: 10.4161/hv.4.1.5257 18441530

[35] Tsvetkov Y Gening ML Kurbatova EA Akhmatova NK Nifantiev NE. Oligosaccharide ligand tuning in design of third generation carbohydrate pneumococcal vaccines. Pure Appl Chem. (2017) 89:1403–1411. doi: 10.1515/pac-2016-1123

[36] Gening ML Kurbatova EA Nifantiev NE. Synthetic analogs of *Streptococcus pneumoniae* capsular polysaccharides and immunogenic activities of glycoconjugates. Russ J Bioorganic Chem. (2021) 47:1–25. doi: 10.1134/S1068162021010076 PMC7980793 33776393

[37] Gening ML Kurbatova EA Tsvetkov YE Nifantiev NE. Development of approaches to a conjugated carbohydrate vaccine of the third generation against *Streptococcus pneumoniae*: the search for optimal oligosaccharide ligands. Russ Chem Rev. (2015) 84:1100–13. doi: 10.1070/RCR4574

[38] Micoli F Romano MR Carboni F Adamo R Berti F. Strengths and weaknesses of pneumococcal conjugate vaccines. Glycoconjugate J. (2023) 40:135–48. doi: 10.1007/s10719-023-10100-3 PMC10027807 36652051

[39] Jansen WT Snippe H. Short-chain oligosaccharide protein conjugates as experimental pneumococcal vaccines. Indian J Med Res. (2004) 119:7–12. 15232153

[40] Weishaupt MW Matthies S Hurevich M Pereira CL Hahm HS Seeberger PH. Automated glycan assembly of a S. pneumoniae serotype 3 CPS antigen. Beilstein J Org Chem. (2016) 12:1440–6. doi: 10.3762/bjoc.12.139 PMC4979738 27559395

[41] Ivanov S Paget C Trottein F. Role of non-conventional T lymphocytes in respiratory infections: the case of the pneumococcus. PLoS Pathog. (2014) 10:e1004300. doi: 10.1371/journal.ppat.1004300 25299581 PMC4192596

[42] Kurbatova EA Akhmatova NK Zaytsev AE Akhmatova EA Egorova NB Yastrebova NE. and Nifantiev NE Higher cytokine and opsonizing antibody production induced by bovine serum albumin (BSA)-conjugated tetrasaccharide related to *Streptococcus pneumoniae* type 3 capsular polysaccharide. Front Immunol. (2020) 11:578019. doi: 10.3389/fimmu.2020.578019 33343566 PMC7746847

[43] Tsvetkov YE Yashunsky DV Sukhova EV Nifantiev NE Kurbatova EA. Synthesis of oligosaccharides structurally related to fragments of *Streptococcus pneumoniae* type 3 capsular polysaccharide. Russ Chem Bull. (2017) 66:111–22. doi: 10.1007/s11172-017-1708-9

[44] Kurbatova EA Akhmatov EA Akhmatova NK Egorova NB Yastrebova NE Romanenko EE. The use of biotinylated oligosaccharides related to fragments of capsular polysaccharides from *Streptococcus pneumoniae* serotypes 3 and 14 as a tool for assessment of the level of vaccine-induced antibody response to neoglycoconjugates. Russ Chem Bull. (2017) 65:1608–16. doi: 10.1007/s11172-016-1488-7

[45] Ananikov VP Eremin DB Yakukhnov SA Dilman AD Levin VV Egorov MP. Organic and hybrid systems: from science to practice. Mendeleev Commun. (2017) 27:425–38. doi: 10.1016/j.mencom.2017.09.001

[46] Akhmatova NK Kurbatova EA Akhmatov EA Egorova NB Logunov DY Gening ML. The effect of a BSA conjugate of a synthetic hexasaccharide related to the fragment of capsular polysaccharide of Streptococcus pneumoniae type 14 on the activation of innate and adaptive immune responses. Front Immunol. (2016) 7:1–11. doi: 10.3389/fimmu.2016.00248 27446078 PMC4919334

[47] Lai Z Schreiber JR. Outer membrane protein complex of meningococcus enhances the antipolysaccharide antibody response to pneumococcal polysaccharide-CRM197 conjugate vaccine. Clin Vaccine Immunol. (2011) 18:724–29. doi: 10.1128/CVI.00053-11 PMC3122513 21450979

[48] Karasartova D Gazi U Tosun O Gureser AS Sahiner IT Dolapci M. Anti-pneumococcal vaccine-induced cellular immune responses in post-traumatic splenectomized individuals. J Clin Immunol. (2017) 37:388–96. doi: 10.1007/s10875-017-0397-3 28488145

[49] Lefeber DJ Benaissa-Trouw B Vliegenthart JF Kamerling JP Jansen WT Kraaijeveld K. Th1-directing adjuvants increase the immunogenicity of oligosaccharide-protein conjugate vaccines related to *Streptococcus pneumoniae* type 3. Infect Immun. (2003) 71:6915–20. doi: 10.1128/iai.71.12.6915-6920 PMC308892 14638780

[50] Reppe K Tschernig T Luhrmann A van Laak V Grote K Zemlin MV. Immunostimulation with macrophage-activating lipopeptide-2 increased survival in murine pneumonia. Am J Respir Cell Mol Biol. (2009) 40:474–81. doi: 10.1165/rcmb.2008-0071OC 18931326

[51] Witzenrath M Pache F Lorenz D Koppe U Gutbier B Tabeling C. The NLRP3 inflammasome is differentially activated by pneumolysin variants and contributes to host defense in pneumococcal pneumonia. J Immunol. (2011) 187:434–40. doi: 10.4049/jimmunol.1003143 21646297

[52] He P Zou Y Hu Z. Advances in aluminum hydroxide-based adjuvant research and its mechanism. Hum Vaccin Immunother. (2015) 11:477–88. doi: 10.1080/21645515.2014.1004026 PMC4514166 25692535

[53] Gupta RK Siber GR. Adjuvants for human vaccines—current status, problems and future prospects. Vaccine. (1995) 13:1263–76. doi: 10.1016/0264-410X(95)00011-O 8585280

[54] Moingeon P Haensler J Lindeberg A. Towards the rational design of Th1 adjuvants. Vaccine. (2001) 19:4363–72. doi: 10.1016/S0264-410X(01)00193-1 11483260

[55] Williams A Flavell RA Eisenbarth SC. The role of NOD-like Receptors in shaping adaptive immunity. Curr Opin Immunol. (2010) 22:34–40. doi: 10.1016/j.coi.2010.01.004 20149616 PMC7038629

[56] Franchi L Núñez G. The NLRP3 inflammasome is critical for alum-mediated IL-1β secretion but dispensable for adjuvant activity. Eur J Immunol. (2008) 38:2085–9. doi: 10.1002/eji.200838549 PMC2759997 18624356

[57] Li H Nookala S Re F. Aluminum hydroxide adjuvants activate caspase-1 and induce IL-1beta and IL-18 release. J Immunol. (2007) 178:5271–6. doi: 10.4049/jimmunol.178.8.5271 17404311

[58] Li H Willingham SB Ting JP-Y Re F Edge C. Inflammasome activation by Alum and Alum’s adjuvant effect are mediated by NLRP3. J Immunol. (2008) 181:17–21. doi: 10.4049/jimmunol.181.1.17 18566365 PMC2587213

[59] Zhang Z Clarke TB Weiser JN. Cellular effectors mediating Th17- dependent clearance of pneumococcal colonization in mice. J Clin Invest. (2009) 119:1899–909. doi: 10.1172/JCI36731 PMC2701860 19509469

[60] Yamamoto N Kawakami K Kinjo Y Miyagi K Kinjo T Uezu K. Essential role for the p40 subunit of interleukin-12 in neutrophil-mediated early host defense against pulmonary infection with *Streptococcus pneumoniae*: involvement of interferon-gamma. Microbes Infect. (2004) 6:1241–9. doi: 10.1016/j.micinf.2004.08.007 15555529

[61] Sun K Salmon SL Lotz SA Metzger DW. Interleukin-12 promotes gamma interferon-dependent neutrophil recruitment in the lung and improves protection against respiratory *Streptococcus pneumoniae* infection. Infect Immun. (2007) 75:1196–202. doi: 10.1128/IAI.01403-06 PMC1828591 17210665

[62] Nakamatsu M Yamamoto N Hatta M Nakasone C Kinjo T Miyagi K. Role of interferon-gamma in Valpha14+ natural killer T cell-mediated host defense against *Streptococcus pneumoniae* infection in murine lungs. Microbes Infect. (2007) 9:364–74. doi: 10.1016/j.micinf.2006.12.003 17314060

[63] Yamada M Gomez JC Chugh PE Lowell CA Dinauer MC Dittmer DP. Interferon-gamma production by neutrophils during bacterial pneumonia in mice. Am J Respir Crit Care Med. (2011) 183:1391–401. doi: 10.1164/rccm.201004-0592OC PMC3114063 21169470

[64] Weber SE Tian H. Pirofski LA CD8+ cells enhance resistance to pulmonary serotype 3 *Streptococcus pneumoniae* infection in mice. J Immunol. (2011) 186:432–42. doi: 10.4049/jimmunol.1001963 PMC3011188 21135172

[65] Lu YJ Gross J Bogaert D Finn A Bagrade L Zhang Q. Interleukin-17A mediates acquired immunity to pneumococcal colonization. PLoS Pathog. (2008) 4:e1000159. doi: 10.1371/journal.ppat.1000159 18802458 PMC2528945

[66] Ma J Wang J Wan J Charboneau R Chang Y Barke RA. Morphine disrupts interleukin-23 (IL-23)/IL-17-mediated pulmonary mucosal host defense against *Streptococcus pneumoniae* infection. Infect Immun. (2010) 78:830–7. doi: 10.1128/IAI.00914-09 PMC2812200 19995896

[67] van Maele L Carnoy C Cayet D Ivanov S Porte R Deruy E. Activation of type 3 innate lymphoid cells and interleukin 22 secretion in the lungs during *Streptococcus pneumoniae* infection. J Infect Dis. (2014) 210:493–503. doi: 10.1093/infdis/jiu106 24577508

[68] Kadioglu A Coward W Colston MJ Hewitt CR. Andrew PW CD4-T lymphocyte interactions with pneumolysin and pneumococci suggest a crucial protective role in the host response to pneumococcal infection. Infect Immun. (2004) 72:2689–97. doi: 10.1128/IAI.72.5.2689-2697.2004 PMC387852 15102777

[69] Malley R Trzcinski K Srivastava A Thompson CM Anderson PW Lipsitch M. CD4+ T cells mediate antibody-independent acquired immunity to pneumococcal colonization. Proc Natl Acad Sci USA. (2005) 102:4848–53. doi: 10.1073/pnas.0501254102 PMC555733 15781870

[70] Trzciński K Thompson CM Srivastava A Basset A Malley R Lipsitch M. Protection against nasopharyngeal colonization by *Streptococcus pneumoniae* is mediated by antigen-specific CD4+ T cells. Infect Immun. (2008) 76:2678–84. doi: 10.1128/IAI.00141-08 PMC2423086 18391006

[71] Wright AK Bangert M Gritzfeld JF Ferreira DM Jambo KC Wright AD. Experimental human pneumococcal carriage augments IL-17A-dependent T cell defence of the lung. PLoS Pathog. (2013) 9:e1003274. doi: 10.1371/journal.ppat.1003274 23555269 PMC3610738

[72] Coffman RL Savelkoul HF Lebman DA. Cytokine regulation of immunoglobulin isotype switching and expression. Semin Immunol. (1989) 1:55–63. 15630959

[73] Zamz S Martinez-Pomares L Jones H Taylor PR Stillion RJ Gordon S. Recognition of bacterial capsular polysaccharides and lipopolysaccharides by the macrophage mannose receptor. J Biol Chem. (2002) 277:41613–23. doi: 10.1074/jbc.M207057200 12196537

[74] Paterson GK Mitchell TJ. Innate immunity and the pneumococcus. Microbiology. (2006) 152:285–93. doi: 10.1099/mic.0.28551-0 16436416

[75] Kadioglu A Weiser JN Paton JC Andrew PW. The role of *Streptococcus pneumoniae* virulence factors in host respiratory colonization and disease. Nat Rev Microbiol. (2008) 6:288–301. doi: 10.1038/nrmicro1871 18340341

[76] van der Poll T Opal SM. Pathogenesis, treatment, and prevention of pneumococcal pneumonia. Lancet. (2009) 374:1543–56. doi: 10.1016/S0140-6736(09)61114-4 19880020

[77] Koppe U Suttorp N Opitz B. Recognition of *Streptococcus pneumoniae* by the innate immune system. Cell Microbiol. (2012) 14:460–66. doi: 10.1111/j.1462-5822.2011.01746.x 22212419

[78] Ferreira LM. Gammadelta T cells: innately adaptive immune cells? Int Rev Immunol. (2013) 32:223–48. doi: 10.3109/08830185.2013.783831 23617235

[79] Tanaka Y Sano S Nieves E De Libero G Rosa D Modlin RL. Nonpeptide ligands for human gamma delta T cells. ProcNatl Acad Sci USA. (1994) 91:8175–9. doi: 10.1073/pnas.91.17.8175 PMC44568 8058775

[80] Sperling AI Cron RQ Decker DC Stern DA Bluestone JA. Peripheral T cell receptor γδ variable gene repertoire maps to the T cell receptor loci and is influenced by positive selection. J Immunol. (1992) 149:3200–207. doi: 10.4049/jimmunol.149.10.3200 1431099

[81] Strominger JL. Developmental biology of T cell receptors. Science. (1989) 244:943–50. doi: 10.1126/science.2658058 2658058

[82] Allison JP. Havran WL.The immunobiology of T cells with invariant gamma delta antigen receptors. Annu Rev Immunol. (1991) 9:679–705. doi: 10.1146/annurev.iy.09.040191.003335 1832874

[83] Havran WL Allison JP. Developmentally ordered appearance of thymocytes expressing different T cell antigen receptors. Nature. (1988) 335:443–5. doi: 10.1038/335443a0 2458531

[84] Bonneville M O’Brien RL Born WK. Gammadelta T cell effector functions: a blend of innate programming and acquired plasticity. Nat Rev Immunol. (2010) 10:467–78. doi: 10.1038/nri2781 20539306

[85] Kalyan S Kabelitz D. Defining the nature of human gammadelta T cells: a biographical sketch of the highly empathetic. Cell Mol Immunol. (2013) 10:21–9. doi: 10.1038/cmi.2012.44 PMC4003173 23085947

[86] Vantourout P Hayday A. Six-of-the-best: unique contributions of gammadelta T cells to immunology. Nat Rev Immunol. (2013) 13:88–100. doi: 10.1038/nri3384 23348415 PMC3951794

[87] Martin B Hirota K Cua DJ Stockinger B Veldhoen M. Interleukin-17- producing gammadelta T cells selectively expand in response to pathogen products and environmental signals. Immunity. (2009) 31:321–30. doi: 10.1016/j.immuni.2009.06.020 19682928

[88] Dieli F Troye-Blomberg M Ivanyi J Fournie JJ Krensky AM Bonneville M. Granulysin-dependent killing of intracellular and extracellular Mycobacterium tuberculosis by Vgamma9/Vdelta2 T lymphocytes. J Infect Dis. (2001) 184:1082–5. doi: 10.1086/323600 11574927

[89] Qin G Mao H Zheng J Sia SF Liu Y Chan PL. Phosphoantigen-expanded human gammadelta T cells display potent cytotoxicity against monocytederived macrophages infected with human and avian influenza viruses. J Infect Dis. (2009) 200:858–65. doi: 10.1086/605413 PMC7110194 19656068

[90] Himoudi N Morgenstern DA Yan M Vernay B Saraiva L Wu Y. Human gammadelta T lymphocytes are licensed for professional antigen presentation by interaction with opsonized target cells. J Immunol. (2012) 188:1708–16. doi: 10.4049/jimmunol.1102654 22250090

[91] Zheng J Liu Y Lau YL Tu W. gammadelta-T cells: an unpolished sword in human anti-infection immunity. Cell Mol Immunol. (2013) 10:50–7. doi: 10.1038/cmi.2012.43 PMC4003172 23064104

[92] Cheng P Liu T Zhou WY Zhuang Y Peng LS Zhang JY. Role of gammadelta T cells in host response against *Staphylococcus aureus*-induced pneumonia. BMC Immunol. (2012) 13:38. doi: 10.1186/1471-2172-13-38 22776294 PMC3524664

[93] Das H Groh V Kuijl C Sugita M Morita CT Spies T. MICA engagement by human Vgamma2Vdelta2 T cells enhances their antigen-dependent effector function. Immunity. (2001) 15:83–93. doi: 10.1016/S1074-7613(01)00168-6 11485740

[94] Bertotto A Gerli R Spinozzi F Muscat C Scalise F Castellucci G. Lymphocytes bearing the gamma delta T cell receptor in acute *Brucella melitensis* infection. Eur J Immunol. (1993) 23:1177–80. doi: 10.1002/eji.1830230531 8477812

[95] Nakasone C Yamamoto N Nakamatsu M Kinjo T Miyagi K Uezu K. Accumulation of gamma/delta T cells in the lungs and their roles in neutrophil-mediated host defense against pneumococcal infection. Microbes Infect. (2007) 9:251–8. doi: 10.1016/j.micinf.2006.11.015 17306586

[96] Kirby AC Newton DJ Carding SR Kaye PM. Evidence for the involvement of lung-specific gammadelta T cell subsets in local responses to *Streptococcus pneumoniae* infection. Eur J Immunol. (2007) 37:3404–13. doi: 10.1002/eji.200737216 PMC2435423 18022862

[97] Cao J Wang D Xu F Gong Y Wang H Song Z. Activation of IL-27 signalling promotes development of postinfluenza pneumococcal pneumonia. EMBO Mol Med. (2014) 6:120–40. doi: 10.1002/emmm.201302890 PMC3936494 24408967

[98] Kirby AC Newton DJ Carding SR Kaye PM. Pulmonary dendritic cells and alveolar macrophages are regulated by gammadelta T cells during the resolution of S. pneumoniae-induced inflammation. J Pathol. (2007) 212:29–37. doi: 10.1002/path.2149 17370296 PMC2970901

[99] Deenen GJ Kroese FG. Kinetics of peritoneal B-1a cells (CD5 B cells) in young adult mice. Eur J Immunol. (1993) 23:12–6. doi: 10.1002/eji.1830230104 7678221

[100] Kroese FG Ammerlaan WA Deenen GJ. Location and function of B-cell lineages. Ann N Y Acad Sci. (1992) 651:44–58. doi: 10.1111/j.1749-6632.1992.tb24592.x 1376060

[101] Martin F Oliver AM Kearney JF. Marginal zone and B1 B cells unite in the early response against T-independent blood-borne particulate antigens. Immunity. (2001) 14:617–29. doi: 10.1016/S1074-7613(01)00129-7 11371363

[102] Margry B Wieland WH van Kooten PJ van Eden W Broere F. Peritoneal cavity B-1a cells promote peripheral CD4+ T-cell activation. Eur J Immunol. (2013) 43:2317–26. doi: 10.1002/eji.201343418 23719868

[103] Sindhava VJ Bondada S. Multiple regulatory mechanisms control B-1 B cell activation. Front Immunol. (2012) 3:372. doi: 10.3389/fimmu.2012.00372 23251136 PMC3523257

[104] Sato T Ishikawa S Akadegawa K Ito T Yurino H Kitabatake M. Aberrant B1 cell migration into the thymus results in activation of CD4 T cells through its potent antigen-presenting activity in the development of murine lupus. Eur J Immunol. (2004) 34:3346–58. doi: 10.1002/eji.200425373 15495164

[105] Vigna AF Godoy LC Rogerio de Almeida S Mariano M Lopes JD. Characterization of B-1b cells as antigen presenting cells in the immune response to gp43 from *Paracoccidioides brasiliensis in vitro* . Immunol Lett. (2002) 83:61–6. doi: 10.1016/S0165-2478(02)00070-6 12057856

[106] Wang Y Rothstein TL. Induction of Th17 cell differentiation by B-1 cells. Front Immunol. (2012) 3:281. doi: 10.3389/fimmu.2012.00281 22973276 PMC3438481

[107] Zimecki M Whiteley PJ Pierce CW Kapp JA. Presentation of antigen by B cells subsets. I. Lyb-5+ and Lyb-5- B cells differ in ability to stimulate antigen specific T cells. Arch Immunol Ther Exp (Warsz). (1994) 42:115–23. 7503644

[108] Zimecki M Kapp JA. Presentation of antigen by B cell subsets. II. The role of CD5 B cells in the presentation of antigen to antigen-specific T cells. Arch Immunol Ther Exp (Warsz). (1994) 42:349–53. 8572891

[109] Berland R Wortis HH. Origins and functions of B-1 cells with notes on the role of CD5. Annu Rev Immunol. (2002) 20:253–300. doi: 10.1146/annurev.immunol.20.100301.064833 11861604

[110] Gao J Ma X Gu W Fu M An J Xing Y. Novel functions of murine B1 cells: active phagocytic and microbicidal abilities. Eur J Immunol. (2012) 42:982–92. doi: 10.1002/eji.201141519 22531922

[111] Zhong X Gao W Degauque N Bai C Lu Y Kenny J. Reciprocal generation of Th1/Th17 and T(reg) cells by B1 and B2 B cells. Eur J Immunol. (2007) 37:2400–4. doi: 10.1002/eji.200737296 17683116

[112] Kobrynski LJ Sousa AO Nahmias AJ Lee FK. Cutting edge: antibody production to pneumococcal polysaccharides requires CD1 molecules and CD8+ T cells. J Immunol. (2005) 174:1787–90. doi: 10.4049/jimmunol.174.4.1787 15699104

[113] Miyasaka T Akahori Y Toyama M Miyamura N Ishii K Saijo S. Dectin-2-dependent NKT cell activation and serotype-specific antibody production in mice immunized with pneumococcal polysaccharide vaccine. PLoS One. (2013) 8:e78611. doi: 10.1371/journal.pone.0078611 24205278 PMC3808275

[114] Miyasaka T Aoyagi T Uchiyama B Oishi K Nakayama T Kinjo Y. A possible relationship of natural killer T cells with humoral immune response to 23-valent pneumococcal polysaccharide vaccine in clinical settings. Vaccine. (2012) 30:3304–10. doi: 10.1016/j.vaccine.2012.03.007 22426326

[115] Su D Shen M Li X Sun L. Roles of γδ T cells in the pathogenesis of autoimmune diseases. Clin Dev Immunol. (2013) 2013:985753. doi: 10.1155/2013/985753 23533458 PMC3600234

[116] Choi YS Baumgarth N. Dual role for B-1a cells in immunity to influenza virus infection. J Exp Med. (2008) 205:3053–64. doi: 10.1084/jem.20080979 PMC2605232 19075288

[117] Haas KM Poe JC Steeber DA Tedder TF. B-1a and B-1b cells exhibit distinct developmental requirements and have unique functional roles in innate and adaptive immunity to *S. pneumoniae* . Immunity. (2005) 23:7–18. doi: 10.1016/j.immuni.2005.04.011 16039575

[118] Popi AF Longo-Maugéri IM Mariano M. An Overview of B-1 cells as antigen-presenting cells. Front Immunol. (2016) 7:138. doi: 10.3389/fimmu.2016.00138 27148259 PMC4827000

